# Flying High: Isometric Strength Training Increases Time of Flight in Junior Elite Trampoline Gymnasts

**DOI:** 10.1002/ejsc.12332

**Published:** 2025-06-10

**Authors:** Natalie Dyas, David Green, Kevin Thomas, Esme Matthew, Ben Young, Glyn Howatson

**Affiliations:** ^1^ Department of Sport, Exercise and Rehabilitation Northumbria University Newcastle‐upon‐Tyne UK; ^2^ UK Sports Institute Manchester UK; ^3^ British Gymnastics Lilleshall National Sports Centre Shropshire UK; ^4^ Water Research Group School of Environmental Sciences and Development Northwest University Potchefstroom South Africa

**Keywords:** performance, recovery, resistance training

## Abstract

The purpose of this study was to assess the effects of a 6‐week isometric training intervention on time of flight (ToF) in elite youth level gymnasts. Fourteen nationally elite youth gymnasts (10 females and 4 males; age = 15 ± 2 years; mass = 55.0 ± 8.2 kg and stature = 163.7 ± 6.5 cm) were recruited. Seven gymnasts in the intervention (INT) group performed a 6‐week isometric squat strength‐training programme, replacing heavy maximal lower limb exercises with three isometric exercises. Seven gymnasts in the control (CON) group performed a standardised strength programme. All gymnasts performed habitual trampoline training. Pre‐ and post‐testing included cycling peak power output, isometric strength, countermovement jumps and 20‐maximum ToF jump tests. Changes in ToF were greater for the INT group (+0.83 s; 2.8%) compared to the CON group (+0.06 s; +0.2%), with a significant group × time interaction effect on ToF (*p* = 0.021). The change in isometric squat peak force at 150° in the INT group (+379 N; 22.4%) was different to the change in the CON group (+78 N; 5.0%), with a significant group × time interaction effect (*p* = 0.032). The isometric intervention provided a sport‐specific training stimulus that was better than traditional heavy maximal resistance training alone for improving isometric strength and ToF in elite trampoline gymnasts.

**Summary**

- Trampolining is an Olympic sport where time of flight (ToF) is an important performance parameter, yet no training‐based interventions on time of flight have been investigated.
- A 6‐week isometric training intervention using joint angles specific to trampoline improved 20‐max ToF performance in gymnasts compared to standard resistance training.
- This study highlighted that improvements in isometric peak force showed transferability to improving ToF on the trampoline and can be used to great effect as an adjuvant intervention alongside traditional resistance training.

## 1 Introduction

Trampolining is an Olympic discipline, which involves routines consisting of acrobatic skills, underpinned by the ability to maximally jump. Time of flight (ToF) is an important objective constituent component of the trampoline scoring system. ToF is commonly measured in trampoline training using maximal jump tests, such as the 20‐maximum jump test (20‐max), which has been shown to be highly reliable (Dyas et al. 2021). Furthermore, very strong and strong positive correlations between countermovement jump (CMJ), F_0_ (theoretical maximal force at null velocity) and 20‐max ToF, respectively, with CMJ F_0_ alone predicting 72% of the variability in 20‐max ToF (Dyas et al. 2023). However, the measure of F_0_ is a theoretical extrapolation of force‐velocity data and is therefore a putative assessment of the theoretical lower limb force‐velocity profile determined through the performance of unloaded and loaded CMJ's.

The CMJ F_0_ is highly determined by the maximal isometric strength and size of the knee extensors (Morales‐Artacho et al. 2018); therefore, it makes the expectation tenable that the maximal isometric strength of the knee extensors could contribute to maximal ToF performance. The ability to maintain knee and hip flexion during the initial contact phase with the trampoline bed is essential to maximise energy transfer between the gymnast and the trampoline. Hip and knee angles on contact with the trampoline have been reported to be between 20° and 40°, respectively (Qian et al. 2020; Rupf and Chapman 2013). Following knee and hip extension, as the trampoline bed depresses and recoils, both hip and knee angles were reported to be less than 10° at the end of the contact phase (Rupf and Chapman 2013). The contact time (and consequent contraction time) for trampoline jumping is longer than normal jumping (0.30–0.35 s). Therefore, trampoline jumping is quasi‐isometric in nature, whereby the gymnast must contact the bed maintaining a small degree of knee and hip flexion and subsequently extend through a relatively small range of motion, over a relatively long period of time for a dynamic movement. These unique kinetic and kinematic characteristics might explain the very strong positive correlations observed between ToF and CMJ F_0_ (Dyas et al. 2023) and highlights the potential for isometric strength training as a suitable stimulus for improving maximal trampoline jumping. There are no studies that have examined the effects of any resistance training intervention on trampoline performance, so further investigation is warranted to understand the application of joint‐angle specific isometric strength training on maximal ToF performance.

Isometric strength training has been shown to induce greater improvements in joint angle specific strength compared to dynamic based strength training (Bridgeman et al. 2018; Folland et al. 2005; Jones and Rutherford 1987; Kanehisa and Miyashita 1983; Kordi et al. 2020). Furthermore, floor‐based dynamic jump performance has also shown improvements following isometric strength interventions (Bimson et al. 2017; Bogdanis et al. 2014). The potential adaptations following an isometric training regimen include increased cross‐sectional area that can be influenced by the volume and duration of the training programme; maximum strength which is predominantly influenced by the intensity of the training and rate of force development that is influenced by the intent to exert force ballistically (Lum and Barbosa 2019). For the improvement of maximal force (the primary physical attribute of interest in this scenario for trampoline athletes), contractions of 80%–100% MVC sustained for 1–5 s at multiple joint angles have been recommended (Lum and Barbosa 2019). Therefore, the purpose of the study was to investigate the effects of a 6‐week isometric training programme on ToF in elite level trampoline gymnasts. It was hypothesised that the sport‐specific joint angles prescribed during isometric training would translate to greater improvements in strength and time of flight performance than traditional resistance training alone.

## 2 Materials and Methods

### 2.1 Subjects

Twenty‐six international and national level gymnasts were recruited from the British Gymnastics (BG) or England national trampoline squads or from high‐level performance clubs within the United Kingdom. Twelve gymnasts withdrew during the course of the study due to injury, unrelated to the intervention, leaving 14 gymnasts (10 females and 4 males; age = 15 ± 2 years; mass = 55.0 ± 8.2 kg and stature = 163.7 ± 6.5 cm). Ethical approval was obtained from Research Ethics committee, and participants provided written informed consent with parental/guardian/carer assent provided in the case of those under the age of 16.

### 2.2 Design

The study used a parallel group design (intervention; INT and control and CON) to examine the effects of a 6‐week isometric strength‐training programme (two sessions per week). Full random allocation of the participants was not possible due to logistical and geographical challenges with conducting an isometric training intervention within the elite trampoline population. Gymnasts were non‐randomly distributed into two groups; controls (CON; 4 females and 3 males) that consisted of England squad gymnasts and intervention (INT; 6 females and 1 male) that consisted of members of the British Gymnastics national squad and national level gymnasts from high performance trampoline clubs. All available gymnasts in the United Kingdom who were of an appropriate age and performance level were recruited for this study. Senior level gymnasts were unable to be recruited due to the timing of their international competition calendar, and younger gymnasts were deemed unsuitable due to their lack of strength and conditioning (S&C) experience. Participants were instructed to refrain from caffeine on the day of testing and arrive in a fed and euhydrated state. All participants were asked to complete a training diary detailing trampoline sessions (number of sessions per week, duration of session, number of contacts and RPE) and S&C sessions (number of sessions per week, duration of session and RPE).

The control group performed their normal habitual trampoline training and were provided with a standardised strength and conditioning (S&C) programme devised by the research team and lead UK Sports Institute S&C coach for the Great Britain trampoline programme. The experimental group also performed their habitual trampoline training and an identical resistance‐training programme to the CON group (Supporting Information S1) but with the inclusion of specific isometric strength exercises, which replaced traditional heavy multi‐joint exercises, such as squats or leg press exercises. The study was completed during the preparation phase leading towards early season competitions.

**FIGURE 1:**
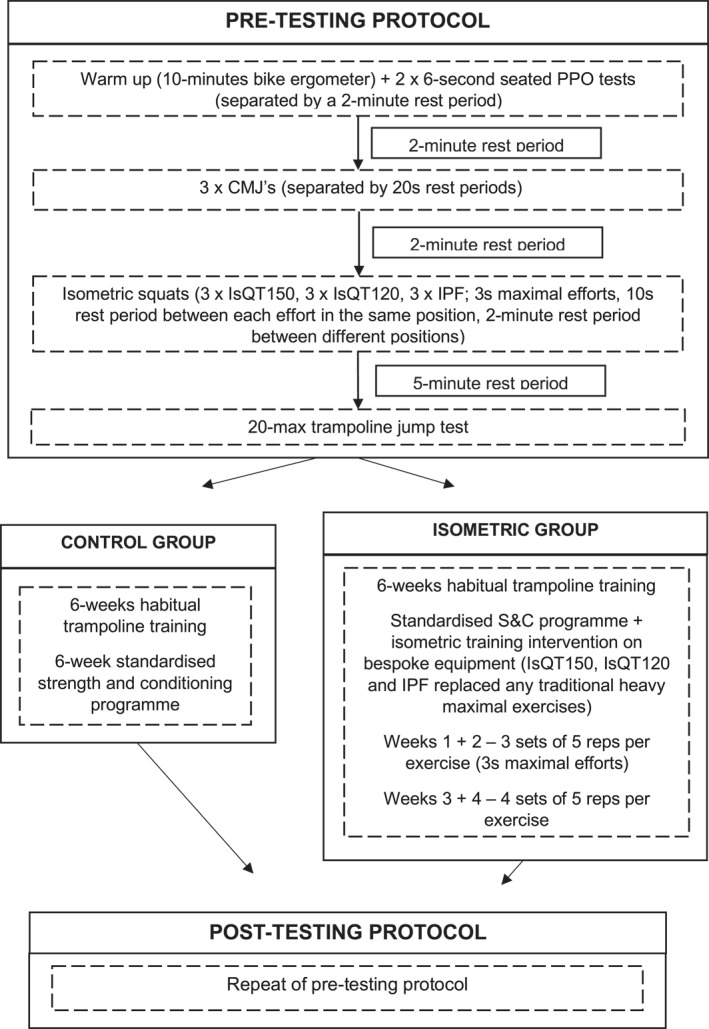
Schematic representation of the testing protocol and intervention design.

Participants performed identical testing pre‐ and post‐intervention (Figure 1), 7 weeks apart. Following stature and body mass measures, a 10 min warm‐up was performed on a cycle ergometer at a rate of perceived exertion (RPE) of 5, on a 0–10 scale (Borg 1998). Participants then performed a battery of tests comprising of seated cycle peak power output tests; CMJ's; isometric squat tests at a variety of different knee and hip angles and 20‐max trampoline jump test. All participants were familiarised with the tests prior to participation. Post‐testing sessions took place 48 h following the final isometric training session.

### 2.3 Methods

#### 2.3.1 Pre‐ and Post‐Testing

##### 2.3.1.1 Seated 6 s Peak Power Output Test

Participants performed two maximal 6 s peak power output (PPO) tests from a stationary start (Wattbike Pro), separated by a 2 min rest period. Resistance was set in line with recommended settings (Wattbike 2020). Strong verbal encouragement and appropriate cues were provided throughout the test, with participants remaining seated throughout. PPO was calculated by the ergometer, and relative peak power output (RPPO) data calculated following the tests. All participants exhibited PPO measures of less than 5% difference between efforts; therefore, mean data from the two efforts were used for analysis.

#### 2.3.2 Countermovement Jumps

Jump testing was performed on a Kistler portable force plate (9290AD, Kistler, Winterhur, Switzerland). Force data were acquired at a sampling frequency of 500 Hz using software packages (Quattro jump, version 1.1.1.4, Kistler, Winterhur, Switzerland). Prior to the test, participants stood in the centre of the force plate whilst a weighing period was performed. Participants were instructed to retain hands on hips and to jump as high as possible for each of the trials. Participants then performed three maximal CMJ's, each separated by 20 s rest. The best trail was used for data analysis. All force data were analysed to determine jump height (cm) calculated from flight time, which was defined as the time from leaving the force plate to first contact back with the force plate.

#### 2.3.3 Isometric Tests

Participants performed three bilateral maximal isometric squats (ISqT) at 3 different angles: knee flexion of 150 (ISqT150); knee flexion of 120 (ISqT120) and standing plantarflexion (IPF). Participants performed each maximal effort for 3 s followed by a 10 s rest between each effort of the same position, followed by a 2 min rest period between each different variation. Participants were instructed to perform the action ‘as hard and as fast as possible’. The best trail in each condition was used for data analysis. For all exercises, feet were positioned hip width apart. Knee angles were measured using a goniometer for the ISqT150 and IsqT120 exercises, with the axis location of the goniometer placed on the lateral epicondyle of the femur, the movement arm along the femur to the greater trochanter and the stationary arm along the fibula to the lateral malleolus. For the IPF exercise, participants were instructed to adopt a ‘soft’ knee position of 170°–180° to avoid hyperextension. The axis location of the goniometer was placed over the lateral malleolus, the stationary arm in line with the head of the fibular and the movement arm in line with the first metatarsophalangeal joint. All participants performed the ISqT tests using a weighted barbell and squat rack whilst positioned on a force plate (Kistler Quattro, 9290AD, Kistler, Winterhur, Switzerland) to obtain peak force measures. Raw force data were acquired at a sampling frequency of 500 Hz (Quattro jump, version 1.1.1.4, Kistler, Winterhur, Switzerland) and analysed offline to calculate peak force (N) and relative peak force (N/kg).

#### 2.3.4 20‐Max Trampoline Jump Test

Following a 5 min rest period, participants performed a 20‐max trampoline jump test on a competition standard trampoline (Eurotramp, Premium 4 × 4, Weilheim an der Teck, Germany). This was connected to a Fédération Internationale de Gymnastique (FIG) approved and calibrated (Ferger et al. 2019) Eurotramp HDTS system (Eurotramp, Germany), with the Eurotramp ToF analysis software (QIRA 1.03, Eurotramp, Germany) used to calculate total ToF the sum total of ToF for each of the 20 consecutive jumps performed during the 20‐max test. Briefly, the force plates under each of the four feet of the trampoline, produce a deformation of the sensor causing a change in light intensity, whereas optoelectronic sensors measure normal force in a one‐dimensional plane (Ferger and Hackbarth, 2017). Scanning is performed at 2 kHz (internally 50 kHz, mean average formation over 25 values), data are provided every 0.5 ms and the sensor has a resolution of less than 0.5 N at an accuracy of 1% (Ferger et al. 2019). Participants started from a static position in the centre of the bed and performed 20 maximal straight jumps, which they all commonly perform during training sessions and has been shown to be highly reliable in trampoline athletes (Dyas et al. 2021).

#### 2.3.5 Training Intervention

**FIGURE 2:**
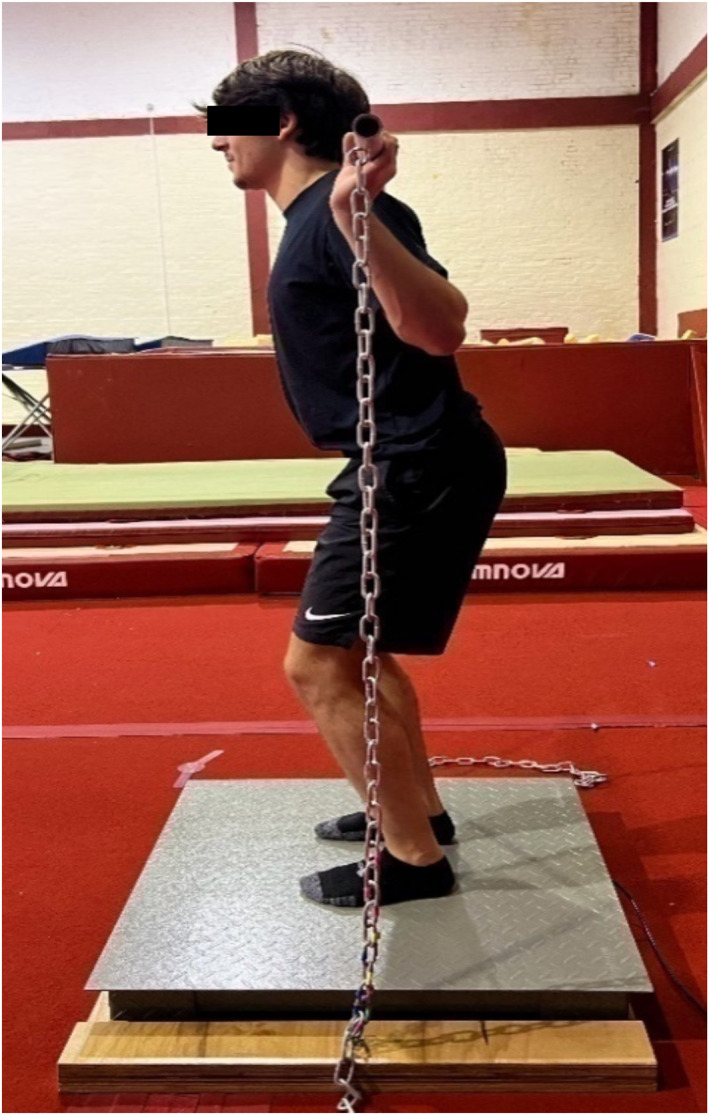
Image of a gymnast performing one of the three joint angles for the isometric intervention. The gymnast is standing on an industrial set of scales, on top of a timber platform, to provide a numerical weight (kg) value (not shown in image) representing the gymnasts effort when performing each isometric contraction. The gymnast is holding a short steel bar on the shoulders, which is attached to eye bolts on the timber platform via steel chains to provide resistance when performing isometric contractions.

The INT group performed three isometric exercise variations; ISqT120, ISqT150 and IPF, which participants performed twice per week for a 6‐week period on bespoke isometric equipment. The isometric exercises replaced any traditional heavy multi‐joint exercises. The bespoke portable equipment (presented in Figure 2) was created as a solution to the lack of access to force plates and squat rack systems in high performance trampoline clubs. The isometric equipment consisted of a timber platform with a recess in the centre for a set of industrial scales (Heavye FW/HF12C Platform Scales and indicator, Hangzhou Heavye Technology Company Ltd., China). The scales acted in place of an expensive portable force plate to provide participants with a numerical marker for each of their maximal isometric efforts. A galvanised steel bar was used to replicate a barbell and consisted of eye bolts on either end. Two heavy duty steel chains with carabiners attached on both ends were used to connect the chains to eye bolts on the timber platform and steel bar. The carabiners allowed for the chain length to be adjusted accordingly for each participant at each of the specific joint angles. A goniometer was used to measure the knee angles of each participant for each IsQT exercise, with coloured tape used to mark each joint angle for each gymnast to allow for ease of use and efficiency during the intervention.

For the intervention, participants performed five maximal repetitions per set, with each maximal effort duration sustained for 3 s with 10 s recovery periods between efforts. Participants were instructed to perform the action ‘as hard and as fast as possible’. The time to complete each set was approximately 60 s, with a total contraction time per set of 15 s. A 2 min recovery was observed between each set and exercise where the participant was inactive. In week 1 and week 2, participants performed 3 sets of 5 repetitions of each exercise. In week 3 and 4, participants performed 4 sets of 5 repetitions, and in week 5 and 6, participants performed 5 sets of 5 repetitions.

All participants recorded training load metrics for the duration of the study, for both trampoline and S&C sessions. Trampoline and S&C training load metrics included number of sessions per week, duration of sessions and session RPE (reported at the end of each session), in line with previous training load research conducted with the British Gymnastics national trampoline programme (Patel et al. 2021). To ascertain maturation status, previously collected data on parental stature was used alongside gymnast age and stature (Khamis and Roche 1994) to calculate percentage predicated adult height (%PAH), in line with previous trampoline gymnastics research (Patel et al. 2021). Gymnast's growth spurt status was defined using %PAH (Pre: < 85%, Circa: 85%–96% and Post: 96% < ) (Parr et al. 2020; Sanders et al. 2017).

### 2.4 Statistical Analysis

Test–retest reliability of the pre‐test variables was assessed using a freely available spreadsheet (Hopkins 2015). Test–retest reliability was calculated using the intra‐class correlation coefficient (ICC), typical error of measurement (TE, raw units) and TE as a log‐transformed (CV, with 95% CI). Calculated ICC values were classified according to the following thresholds: 0.9 nearly perfect; 0.7–0.9 very large; 0.5–0.7 large, 0.3–0.5 moderate and 0.1–0.3 small (Hopkins 2002). A CV of ≤ 5% was considered as good between‐session reliability for performance tests (Atkinson and Nevill 2000; Buchheit et al. 2011). All repeatability data are presented in Table 1. Participants performed one 20‐max test during the pre‐testing protocol and post‐testing protocol; however, the reliability of the 20‐max trampoline test has previously been reported across range of national and international level trampoline gymnasts (ICC = 0.96; TE = 0.41, CV% = 1.3, Dyas et al. 2021).

**TABLE 1:** Reliability of the testing variables.

Reliability statistics (95% CI)			
	ICC	TE	CV%
Cycle RPPO (W/kg)	0.98 (0.93–0.99)	0.16 (0.12–0.28)	4.2 (2.9–7.2)
CMJ height (cm)	0.98 (0.92–0.99)	0.17 (0.12–0.28)	2.5 (2.9–7.2)
PF ISQT120 (N)	0.92 (0.82–0.97)	0.30 (0.23–0.43)	5.7 (4.1–9.1)
RPF ISQT120 (N/kg)	0.89 (0.75–0.96)	0.35 (0.28–0.51)	5.7 (4.1–9.1)
PF IsQT150 (N)	0.97 (0.92–0.99)	0.19 (0.15–0.28)	4.4 (3.2–7.0)
RPF IsQT150 (N/kg)	0.97 (0.92–0.99)	0.20 (0.15–0.29)	4.4 (3.2–7.0)
PF IPF (N)	0.98 (0.94–0.99)	0.16 (0.12–0.26)	7.9 (3.2–20.1)
RPF IPF (N/kg)	0.86 (0.68–0.95)	0.40 (0.31–0.58)	7.9 (3.2–20.1)

Abbreviations: CIs, confidence intervals; CMJ, countermovement jump; CV, coefficient of variation; ICC, intra‐class correlation coefficient; PF IPF, peak force plantar flexion; PF IsQT120, peak force isometric squat 120° knee flexion; PF IsQT150, peak force isometric squat 150° knee flexion; RPF IPF, relative peak force plantarflexion; RPF IsQT120, relative peak force isometric squat 120° knee flexion; RPF IsQT150, relative peak force isometric squat 150° knee flexion; RPPO, relative cycle peak power output; TE, typical error.

All *t*‐test and ANOVA analyses were performed with SPSS 27 (IBM, New York, USA), and the threshold for statistical significance was *p* ≤ 0.05. Data are reported as mean ± standard deviation. For suitable tests, alongside absolute values, data are normalised to body mass. Baseline measures were compared between groups using independent *t*‐tests. Mixed factorial, 2 × 2 (Group; INT, CON, by Time; pre‐, post‐) ANOVAs were used to assess the differences between groups and the effect of the isometric training intervention. Effect sizes were calculated using partial eta‐squared (*η*_*p*_^2^; small = 0.010–0.059; medium = 0.060–0.149 and large ≥ 0.150), (Cohen, 1988). Homogeneity of variance was assessed via Levene's statistic and, where violated, Welch's adjustment was used to correct the F‐ratio.

## 3 Results

**FIGURE 3:**
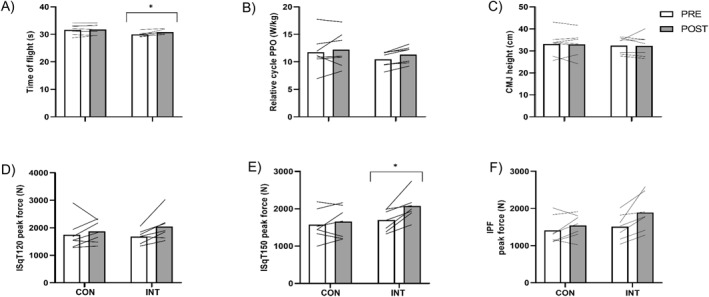
Pre‐ and post‐group responses (bars) and individual responses (line) for the control and intervention groups for time of flight (Panel A), relative cycle PPO (Panel B), CMJ height (Panel C), isometric peak force at 120° knee flexion (Panel D), isometric peak force at 150° knee flexion (Panel E) and peak force plantarflexion (Panel F). Significant group x time interactions (*p* < 0.05) were supported by post hoc tests revealing time of flight, isometric squat absolute and relative peak force improved in the intervention group but not the control group.

No differences were observed between baseline measures of mass (55.9 ± 8.0 vs. 54.0 ± 8.9 kg), age (15 ± 2 vs. 14 ± 1 year), stature (163.1 ± 6.8 vs. 164.4 ± 6.7 cm) or maturation status (98.2 ± 3.2 vs. 97.4 ± 1.8%). Most participants were post‐growth spurt, except for two gymnasts in the INT group and one gymnast in the CON group. Training load metrics for trampoline specific sessions or S&C sessions did not differ between CON and INT groups (number of training sessions = 3.0 ± 0.9 vs. 3.0 ± 1.1; total session duration = 186 ± 90 vs. 194 ± 106; trampoline contacts 225 ± 95 vs. 233 ± 86 and RPE 5.4 ± 1.2 vs. 5.3 ± 1.3). Individual responses within each group for each of the assessments are displayed in Figure 3, with the average percentage changes within groups displayed in Table 2.

**TABLE 2:** Pre‐, post‐ and percentage (%) change of measured physical dependent variables for the isometric and control groups.

	Intervention group			Control group			ANOVA	
	Pre‐	Post‐	% change (range)	Pre‐	Post‐	% change (range)	Time × group interaction (*p* =)	Effect size
Total ToF (s)	29.95 ± 0.84	30.78 ± 0.86^a^ ^,^ ^b^	2.8 (1.4–6.9)	31.66 ± 1.88	31.72 ± 1.84	0.2 (−1.8–3.2)	0.02	0.38
Cycle RPPO (W/kg)	10.4 ± 1.4	11.3 ± 1.5^a^	8.7 (3.2–13.8)	11.7 ± 3.3	12.2 ± 3.2	5.2 (−16.2–25.2)	0.51	0.04
CMJ height (cm)	32.4 ± 3.8	32.3 ± 4.9	0 (−7.3–15.9)	33.1 ± 5.7	32.9 ± 5.6	−0.6 (−12.7–4.5)	0.90	0.01
PF ISqT120 (N)	1684 ± 245	2047 ± 499^a^	21.5 (–3–47.4)	1746 ± 561	1874 ± 402	7.3 (−21.3–29.9)	0.24	0.11
RPF ISqT120 (N/kg)	31.7 ± 7.0	39.3 ± 10^a^	24.0 (2.4–46.8)	30.8 ± 5.8	33.2 ± 6.5	7.8 (−22.8–28.2)	0.12	0.19
PF ISqT150 (N/kg)	1693 ± 288	2072 ± 358^a^ ^,^ ^b^	22.4 (4.7–38.9)	1575 ± 404	1653 ± 427	5.0 (−12.9–31.9)	0.03	0.39
RPF ISqT150 (N/kg)	30.7 ± 5.2	39.0 ± 7.2^a^ ^,^ ^b^	27.0 (4.6–38.9)	28.2 ± 5.9	29.6 ± 7.1	5.0 (−11.3–31.9)	0.02	0.36
PF IPF (N)	1508 ± 348	1893 ± 492^a^	25.5 (−13.4–35.2)	1411 ± 366	1542 ± 318	9.3 (−12.3–35)	0.10	0.21
RPF IPF (N/kg)	28.2 ± 6.2	35.0 ± 6.6^a^	24.0 (−4.7–59.4)	25.2 ± 5.1	27.6 ± 5.5	9.5 (−13.4–35.2)	0.10	0.21

Abbreviations: CMJ, countermovement jump; PF IPF, peak force plantar flexion; PF IsQT120, height, peak force isometric squat 120° knee flexion; PF IsQT150, peak force isometric squat 150° knee flexion; RPF IPF, relative peak force plantarflexion; RPF IsQT120, relative peak force isometric squat 120° knee flexion; RPF IsQT150, relative peak force isometric squat 150° knee flexion; RPPO, relative cycle peak power output; ToF, total time of flight.

^a^ Significant difference from a baseline value (*p* < 0.05).

^b^ Significant group × time interaction effect (*p* < 0.05).

### 3.1 Time of Flight

At baseline, the mean ToF for the groups at baseline was 31.656 ± 1.883 s and 29.945 ± 0.836 s for the CON and INT, respectively, which was not statistically different (*p* = 0.06). The change in ToF in the INT group was greater than the change in the CON group, with a significant group × time interaction effect on ToF (F_(1,12)_ = 7.07 and *p* = 0.021). Individual responses within each group for ToF are displayed in Figure 3, with the INT group increasing ToF on average by 2.8% compared to 0.2% in the CON group (Table 2).

### 3.2 Isometric Assessments

At baseline, there were no differences for any isometric values between the CON and INT groups. The change in ISqT150 peak force in the INT group (+379 N; +22.4%) was greater than the change in the CON group (+78 N; +5.0%), with a group × time interaction effect (F_(1,12)_ = 5.87 and *p* = 0.032). In addition, the increase in ISqT150 relative peak force in the INT group (+8.3 N/kg; +27%) was greater than the change in the CON group (+1.4 N/kg; +5%), with a group × time interaction (F_(1,12)_ = 6.70 and *p* = 0.024).

There were no interaction effects across the other isometric assessment metric (*p* > 0.05, Table 2). There were main effects of time for ISqT120 peak force (F_(1,12)_ = 6.75 and *p* = 0.023), ISqT120 relative peak force (F_(1,12)_ = 10.90 and *p* = 0.006), IPF peak force (F_(1,12)_ = 13.30 and *p* = 0.003) and IPF relative peak force (F_(1,12)_ = 16.57 and *p* = 0.002), with all variables improving from pre‐ to post‐intervention (Table 2).

### 3.3 Cycle RPPO and CMJ Height

At baseline, there were no differences for either cycle RPPO or CMJ height between the CON and INT groups. There were no significant interaction effects across either cycle RPPO or CMJ height (*p* > 0.05). However, there was a main effect of time where cycle RPPO increased (F_(1,12)_ = 5.18 and *p* = 0.042).

## 4 Discussion

The aim of this study was to assess the effectiveness of a 6‐week isometric strength training intervention on ToF in elite level youth gymnasts. This is the first study to assess the effectiveness of any strength‐based intervention on ToF in trampoline gymnasts and showed that an adjuvant 6‐week isometric training programme incorporated into routine strength sessions improved 20‐max ToF performance compared to a standard resistance‐based strength programme alone. Improvements were also shown for the INT group for ISqT150 peak and relative peak force, with both measures displaying large effect sizes. No changes were evident from pre‐ and post‐testing for all measures in the CON group. The study also highlighted the feasibility of using bespoke isometric equipment that can be successfully embedded within clubs to deliver performance improvements, without the need for expensive S&C equipment. Importantly, this study showed that improvements in isometric peak force at sport‐specific joint angles can improve isometric peak force that translates to improving ToF performance on the trampoline.

The study showed that two weekly isometric strength training sessions for a 6‐week period improved 20‐max ToF values in an elite group of gymnasts. Although isometric training has been shown to improve elements of dynamic performance (Lum et al. 2021), this is the first study to demonstrate the translation of isometric resistance exercise to an elite cohort of this nature. In comparison, the CON group who performed a standardised S&C programme exhibited no ToF improvements. The INT group showed an average ToF increase of 2.8%, whereas the CON group displayed no ToF improvements, highlighting that specific isometric training enhances ToF beyond a standardised traditional S&C stimulus. The 20‐max test has been previously shown to have excellent test–retest reliability (Dyas et al. 2021), across a large cohort of elite level trampoline gymnasts (ICC = 0.96; TE = 0.41 and CV% = 1.3). Consequently, in that context, the changes in the INT group are a meaningful improvement in ToF that is unlikely to be attributed to measurement error and therefore likely very beneficial for elite athletes.

All ISqT120, plantarflexion and cycle RPPO variables significantly improved from pre‐to post‐testing across the INT group. Apart from IsQT120 absolute peak force, which exhibited a medium effect size, all other isometric measures displayed large effect sizes. However, alongside total 20‐max total ToF, the only other variables that exhibited significant interaction effects for the INT group were ISqT150 peak force and relative peak force. The joint angles used in the training intervention in this study were chosen as they covered a range of lower limb joint angles the gymnasts are reported to experience on contact with the trampoline. However, the ISqT150 resembles the closest knee flexion angle to that reported on initial contact (20°–40°) with the trampoline bed during maximal jumping (Qian et al. 2020; Rupf and Chapman 2013). It is essential that the gymnast maintains knee and hip angles on initial contact with the trampoline bed, to maximise energy transferral between themselves and the trampoline to translate to a greater ToF. This demands the production of high levels of force in a quasi‐isometric muscle action to maintain the height of the centre of mass, and subsequently extend the knee and hip, across a contact time of 0.30–0.35 s. Therefore, the increase in ToF is likely contributed to by the increases in ISqT150 shown in the INT group.

The significant gains in ToF and isometric peak force values were exhibited in response to a relatively short 6‐week intervention. To increase maximum isometric strength, it has been proposed that multiple joint angles or angle‐specific isometric contractions should be performed maximally with a sustained contractions between 1 and 5 s and between 30 and 90 s total contraction time per session (Lum and Barbosa 2019). In this study, isometric contractions performed were maximal, with feedback provided via the training device. This provided gymnasts and S&C coaches with an indication of the effort throughout the intervention. However, the current study utilised a greater total contraction time of 135–225 s per session compared to 30–90 s total contraction time per session recommended in a review by Lum and Barbosa (2019) for improving maximal isometric strength. Although comparable data in youth gymnasts are not available, similar improvements in ISqT120 peak force have been seen across a similar 6‐week isometric intervention in adult national floorball players (Lum et al. 2021). Lum et al. (2021) reported average improvements of 427 N in the intervention group and 94 N in the control compared to an average increase of 363 N in the INT group and 128 N in the CON in the current study. Some improvements in the CON group were expected because they continued with their habitual trampoline and strength training and made physical improvements in some performance metrics. Nonetheless, performing a short 6‐week supplementary isometric intervention to improve isometric peak force at the trained joint angles had greater transferability to a sport‐specific performance test in the INT group than traditional strength training.

One important point is worth highlighting; all participants in the INT group exhibited increases in 20‐max total ToF (1.4%–6.9%) and increases in cycle RPPO, ISqT120, IsQT150 and plantarflexion metrics. However, only three gymnasts improved CMJ height in the INT group, with the same number improving in the CON group. The individual responses in the CON group for all metrics were mixed for all ToF and other performance metrics. Two gymnasts in the INT group and one in the CON group were circa‐growth spurt during the study, with all other post‐growth spurt. The notion of synergistic adaptation has been reported as a limitation of training interventions in younger athletes, whereby adaptations resulting from interventions can complement the naturally developing adaptations of growth and maturation (Lloyd et al. 2016; Moeskops 2020; Radnor et al. 2017). However, only three out of the 14 gymnasts in the current study were circa‐growth spurt, with no augmented responses identified in those gymnasts compared to those post‐growth spurt. Not only has the isometric intervention shown improvements in joint angle specific peak force across all gymnasts and transferability to a trampoline specific test, the ease of use and safety of this approach also adds to its appeal compared to traditional heavy lifting exercises. The nature of the study meant that we were not able to attribute these adaptations to specific physiological mechanisms. However, given the training stimulus provided (joint‐specific peri‐maximal contractions of ∼3 s, with maximal intent), it is likely that improvements in maximal strength and rate of force development (RFD) could be responsible for the performance improvements. The responses observed in the current study support previous work (Lum and Barbosa 2019) that concluded joint‐specific isometric training can be translated to dynamic sports performance, and high intensity contractions will result in improved neuromuscular adaptations through increased strength and RFD.

A limitation of the study was the lack of true randomisation of the INT and CON groups. In this study, it was not possible to randomly allocate participants due to logistical reasons. Only gymnasts at selected high‐performance clubs were recruited in the INT group. Participants were allocated to the groups based on their training venue and squad membership. This limitation was necessary to allow for as many participants as possible to complete the INT at selected high‐performance clubs where S&C support was available. This does bring in to question the potential limitation surrounding maturation, biological sex and competition level; however, where differences were observed, the response to training was directionally the same. The CON group also had a prescribed S&C programme; therefore, small differences between exercise programming were possible. Despite these challenges, the study has a very high degree of external validity, given not all athletes are the same and the training they do might vary to some extent. Further, options were provided to clubs to account for possible differences in equipment availability in CON. For example, leg press and back squat were two options provided for bilateral triple leg extension exercises for the CON group. Although both exercises train similar lower limb muscles, leg press requires less muscle activity towards stabilising the body compared to the back squat (Wirth et al. 2016). Anecdotally, some INT gymnasts had minor difficulties counteracting anterior‐posterior sway on the isometric equipment, particularly when performing the maximal plantarflexion contractions. This is less likely to occur if performing exercises on a squat rack; however, the isometric equipment provided an opportunity to perform isometric exercises within clubs.

## 5 Conclusion

Time of flight is a critical performance index that is quantified and scored in competition, such that greater time of flight translates to higher scores. This study showed that a 6‐week adjuvant isometric training programme improved ToF performance compared to a standard resistance‐based strength programme alone in elite gymnasts. Improvements were also observed in isometric absolute and relative peak force at 150° of knee flexion. Training at this joint angle is more task‐specific when translating to improved ToF performance and more closely replicates the knee joint angle employed by many gymnasts on contact with the trampoline bed. This study provides new evidence that improving lower limb maximal isometric capabilities at specific joint angles can improve ToF. Collectively, the implementation of isometric strength training in elite trampoline gymnasts is not only a feasible and tolerable training method but also highly effective in its transfer to sport specific performance indices. The improvements in ToF showed the ability for specific isometric training to enhance maximal ToF, with the view that this will show transfer to in‐jump ToF allowing gymnasts to begin at, and maintain, greater heights throughout routines. Similarly, the increase in ToF could also transfer through to acrobatic skills during routines by allowing more ‘airtime’ to perform these skills and thereby increasing the potential for Olympic medal success in trampolining. Previous work showed maximal isometric lower limb force to be an important determinant of maximal ToF (Dyas et al. 2023). The data from the current study build on this previous knowledge to show this a trainable quality that can improve maximal ToF when incorporated alongside traditional strength training and provides practitioners and coaches with a practical, cost‐effective gym‐based method to increase performance indices in elite trampoline gymnasts.

## Ethics Statement

Ethical approval was obtained from Northumbria University Research Ethics committee.

## Conflicts of Interest

The authors declare no conflicts of interest.

## Supporting information

Supporting Information S1
