# Supplementary material for: Flying High: Isometric Strength Training Increases Time of Flight in Junior Elite Trampoline Gymnasts
Source: Eur J Sport Sci. 2025 Jun 10;25(7):e12332. doi: 10.1002/ejsc.12332 (PMC12149603; doi:10.1002/ejsc.12332)
Supplement: Supplementary file 1 — Supporting Information S1 [file EJSC-25-e12332-s001.pdf]

Session 1			Week 1			Week 2			Week 3			Week 4			Week 5			Week 6		
			Sets	Reps	Load	Sets	Reps	Load	Sets	Reps	Load	Sets	Reps	Load	Sets	Reps	Load	Sets	Reps	Load
1a	Double Leg Drop Landing		3	3		3	4		3	5		3	3		3	4		3	5	
1b	Pogos			10			12			14			16			18			20	
2a	Double Leg Triple Extension: (E.g. Goblet Squat)		4	6		4	7		4	8		4	6		4	7		4	8	
2b	Push: (E.g. Press Up)			10			11			12			13			14			15	
3a	Single Leg Tiple Extension: (E.g. Barbell [illegible] Step Up)		4	8		4	10		4	12		4	8		4	10		4	12	
3b	Pull: (E.g. Lat Pulldown)			8			8			10			10			12			12	
4a	Hinge: (E.g. Good Morning)		4	10		4	11		4	12		4	10		4	11		4	12	
4b	Hamstring: (E.g. Stability Ball Hamstring Curl)			10			12			14			16			18			20	
5a	Calf 1: (E.g. Plea Calf Raises)		4	15		4	18		4	21		4	24		4	27		4	30	
5b	Foot & Ankle 1: (e.g. Banded dorsi flexion)			15			18			21			24			27			30	

Session 2			Week 1			Week 2			Week 3			Week 4			Week 5			Week 6		
			Sets	Reps	Load	Sets	Reps	Load	Sets	Reps	Load	Sets	Reps	Load	Sets	Reps	Load	Sets	Reps	Load
1a	Countermovement Jump		3	3		3	4		3	5		3	3		3	4		3	5	
1b	Pogos			10			12			14			16			18			20	
2a	Double Leg Triple Extension: (E.g. Goblet Squat)		4	6		4	7		4	8		4	6		4	7		4	8	
2b	Push: (e.g. Shoulder Press)			10			11			12			13			14			15	
3a	Single Leg Tiple Extension: (E.g. Box Squat)		4	8		4	10		4	12		4	8		4	10		4	12	
3b	Pull: (e.g. Inverted Row)			8			8			10			10			12			12	
4a	Hinge: (e.g. Good Morning)		4	10		4	11		4	12		4	10		4	11		4	12	
4b	Hamstring: (E.g. Nordics)			5			5			5			5			5			5	
5a	Calf 2: (E.g. Single Leg Straight Leg Calf Raises)		4	15		4	18		4	21		4	24		4	27		4	30	
5b	Foot & Ankle 2: (Banded Eversions)			15			18			21			24			27			30	

	Double Leg Triple Extension	Single Leg Triple Extension	Hinge	Hamstring	Calf	Foot & Ankle	Push	Pull
1	Wall Ski Sit (1rep =3s)	Step Up	Hip Thurst	DL Stability Ball Curls	DL/SL Straight Knee Standing	Banded Eversions	Press Up	Inverted Row
2	Goblet Squat	Box Squat	Good Morning	SL Stability Ball Curls	DL/SL Bent Knee Standing	Banded Inversions	DB Bench press	Pull/ Chin Up
3	Hexbar Deadlift	Lateral Box Step Up	Romanian Deadlift	SL Foam Roller Curls	DL Plea Calf Raises	Banded Dorsi Flexions	Barbell Bench Press	Bench DB Row
4	Back Squat	Bulgarian Split Squat	Reverse Hyperextension	DL Hamstring Curl Machine	DL/SL Seated Calf Raise	Forefoot Balances	Shoulder Press	Prone Row
5	Leg Press	Leg Press		Nordics (5 reps)	DL/SL Leg Press	Y Balance	Dips	Lat Pulldown

Low Load Trunk			
3x pw	Exercise	Reps	Sets
1	V Sits	20s	3
2	Side Plank Dips L	20s	3
3	Alekna	20s	3
4	Side Plank Dips R	20s	3
5	Ab Cycles	20s	3
6	Plank Shoulder Taps	20s	3
Increase sets up to 4 or duration up to 25/30s if you want to progress			

Dynamic Motor Control			
3x pw	Exercise	Reps	Sets
1	Banded Parlov Press	20s	3
2	Banded Kneeling Overhead Parlov Press	20s	3
3	Banded Kneeling Woodchops - High to Low	20s	3
4	Banded Standing Woodchops - Low to high	20s	3
5	Reverse Hyperextensions off bench	15r	3
6	Lateral Crunches using a stability ball	15r	3
Control range and tempo throughout - progress my increasing load			

Notes:
Weeks 1 to 3 progress via volume, then in week 4 when the reps drop down increase the load
When the volume stays the same either maintain the weight or increase
When the volume always increases keep the load the same
The Nordics are an eccentric exercise to failure, they don't need to increase intensity as they will always be maximal

DL	Double Leg
SL	Single Leg
DB	Dumbbell
